# Report from the Melanoma Independent Board First Melanoma MIB Conference, 21–22 October 2013

**DOI:** 10.3332/ecancer.2014.440

**Published:** 2014-06-30

**Authors:** A Testori, P Ascierto, V Chiarion Sileni, F De Lorenzo, PG Pelicci, CR Rossi

**Affiliations:** 1 European Institute of Oncology, Via Ripamonti 435, Milan 20141, Italy; 2 Istituto Nazionale dei Tumori di Napoli, Italy; 3 Istituto Oncologico Veneto, Padova, Italy; 4 Federazione Italiana delle Associazioni di Volontariato in Oncologia, Italy

**Keywords:** melanoma, cost-effective treatment, treatment guidelines, new therapies

## Abstract

The Melanoma Independent Board (MIB) held its first conference from 21 to 22 October, 2013, in Rome, Italy. Like the MIB itself, the conference brought together specialists from all aspects of cancer care: doctors, patient associations, journalists, and representatives from local government, hospitals, and pharma to encourage an interdisciplinary discussion on the future of melanoma. It was hoped that the conference would be an opportunity for all participants to see and understand each other’s points of view. In memoriam of melanoma pioneer Natalie Cascinelli, the conference focussed on innovation and sustainability as well as the latest drug developments.

## Introduction

Since being considered one of the most aggressive types of cancers in the 1950s, there have been significant advances in melanoma knowledge, and it is now often successfully treated through surgery. However, there are still a number of challenges associated with melanoma treatment, including continued differing viewpoints on surgical approaches and few available alternatives to surgery. Coupled with an ever-increasing rate of melanoma worldwide, there is a significant need for research in melanoma care.

The past few years have seen an increase in alternative treatments for melanoma, but with these new treatments, there is a need to address budgetary and organisational constraints to research and development. Pharmaceutical companies, while undertaking a large portion of research and development, have limited resources and are facing increasing pressures in trying to adhere to regional, national, and international regulations for clinical trials and drug approval. Personalised medicine, while an important step forward in cancer treatment, is also very expensive since treatments are tailored to individual patients. In addition, hospitals carrying out the treatments have their own financial constraints, which must be considered when using some very expensive drugs [1–7].

The Melanoma Independent Board (MIB) was established in 2012, and is a network for doctors, patient associations, journalists, and representatives from local government, pharma, and AIFA, the Italian Medicines Agency. While made up primarily of Italians, one of its aims is to grow internationally into the rest of Europe and worldwide. It is hoped that the diverse range of members can allow for a deeper understanding of all the challenges associated with melanoma care. Thus, with this shared knowledge, the group will hopefully be able to establish guidelines and recommendations for the use of new drugs coming into the clinic. As one can tell from its name, it is independent of pharmaceutical influence, and any recommendations made will be in the best interest of all.

The MIB held its first conference in Rome, Italy, from 21 to 22 October 2013. The conference was an opportunity to discuss and learn from each other’s point of view. The specialists from Italy and the rest of Europe looked at the future of melanoma care. Held in memoriam of Natalie Cascinelli, the focus of the conference was broken down into three main categories: 1) strategic, 2) scientific, and 3) clinical issues. These three topics were also the categories of parallel workshops held in the second half of the meeting. Of special importance were sustainability, cost-effectiveness, and greater national and international collaboration. In addition, some recent treatment advances were discussed.

## Main sessions

### First session

The first day was broken down into three main sessions, with a discussion period at the end of each session. Alessandro Testori, from the European Institute of Oncology (IEO), Milan, started off with a welcome to the attendees on behalf of the MIB and reminded everyone of the goals of the conference to enhance understanding and knowledge between the various branches of cancer carers and also keep in mind the challenges associated with development and organisation. Paolo Foggi continued the welcome on behalf of the AIFA (Italian Medicines Agency) and expressed his eagerness to have a dialogue with different experts, and he reminded everyone of the issues of sustainability that melanoma care is facing.

Dr Testori and Nicola Mozzillo from the Istituto Nazionale Tumori in Naples, Italy, started the first session with a brief summary of the history of melanoma care and highlighted the major advances that were made in the 1960s. The turning point in the understanding and treatment of melanoma can be attributed to two early international collaborations. In the first, the relationship between clinical and histological aspects was studied. The results of the international research led to a better understanding of what part of a lesion needs to be excised and also the development of the ABCD(E) classification to help dermatologists classify lesions. Around the same time, a World Health Organisation melanoma programme established in 1965, and eventually involving 70 institutions worldwide addressed to main questions: what is adequate surgery and what is the role of post-operative adjuvant therapy? The results of the study proved that differing techniques among Europeans and Americans produced the same results. Natalie Cascinelli, to whom the meeting was dedicated, was involved in much of these early studies, especially in the determination of adequate surgery needed for melanoma excision. Cascinelli’s work and results are still relied upon by melanoma doctors today.

Sara Gandini, senior staff scientist at the IEO in Milan, Italy, provided a further introduction into the evolution of melanoma, but this time from an epidemiological perspective. Cutaneous melanoma accounts for about three quarters of all skin cancer deaths. There are 88,000 new cases diagnosed in Europe each year and 21,000 deaths [8]. Although Australia has the highest rate of melanoma incidence, both the USA and Europe saw a threefold increase in melanoma in a 35-year period from 1970, and it is believed that it will continue to increase over the next two decades. Risk factors, such as sun-bed use, and incidence in socio-economic status were also discussed.

The next talk, by Francesco de Lorenzo, president of the European Cancer Patient Coalition (ECPC), turned the focus onto the patient experience. He started his talk with the growth of the Italian patient group, FAVO, and some of the major advancements the group have made. In particular, the group has decreased the time needed to declare cancer as a disability and they have worked to improve drug access among regions of Italy. He then shifted his focus to the ECPC, which represents all cancers and aims to promote timely access to prevention, screening, early diagnosis, treatment, and care for all cancer patients [9].

The ECPC represents over 300 groups in 44 countries. The melanoma section has developed a new document entitled ‘The melanoma white paper: Reshaping EU Healthcare for Melanoma Patients’, which is for all European patients [10]. He concluded his talk reiterating his group’s dedication to melanoma care. Melanoma is an underserved cancer, and the ECPC hopes to help all patients across Europe receive the best care.

Next, the challenges faced by pharmaceutical companies were highlighted by Maurizio de Cicco, the vice president of Farmindustria. These challenges include the rising costs of research and development, in part due to the growth of personalised medicine, as well as expected cuts to the industry. He stated that pharma spending is about 14% of total health-care public expenditure, but in the last few years, pharma has been subjected to 30% of the total cuts. The cost of drugs is minimal compared with the total health-care spending. Additional challenges include a further reduction in expected finances for most pharma companies as well as the long time and cost involved in drug approval. In Italy, it takes about 2 years for a drug to reach patients from initial EU approval. Even then there are a number of restrictions on the use of the drug. For example, in Italy, there is not only the national health-care system that must be considered, but many regions have their own specific systems. Dr de Cicco presented some money-saving ideas such as cost sharing, (where there is a price rebate on the first therapy cycle for all patients), risk sharing (where there is 50% reimbursement of the first therapy cycles for non-responder patients at the first re-evaluation), and payment by results (where there is a total reimbursement of the first therapy cycles for non-responder patients at the first re-evaluation).

Ruggero Cadossi discussed some of the challenges associated with gaining approval for combination treatments that use both a drug and a device. New devices must have clinical data to support their safety and to prove their need compared with other therapies. He gave an example of electroporation, which combines both a device and a drug. Such combination treatments have very challenging and expensive regulatory approval processes.

Luciano Onder, a medical journalist for Radio Television Italiana (RAI), looked specifically at the health-care system in Italy. He stated that there are too many small hospitals still functioning, which is just one of the problems of the Italian health system that needs to be addressed. There are too many small hospitals with inadequate services (outdated) with the increasing cost of new drugs. We need a program to decide who gets what and when.

The topic of money saving was continued by Andrea Messori from ESTAV, with a talk on dealing with the cost of new drugs. When looking at a new drug, benefits are converted to ‘quality-adjusted life years’ (QALYs). A very simplified means of assessing QALYs assumes that each month of life gained is valued at €5000 (= 60,000/year). Orphan drugs were also highlighted as the cost of these drugs is inversely proportional to the number of cases. However, the cost of orphan drugs should be reduced if the drug substance is cheap or if clinical trials have been carried out by independent researchers. In these two cases, a hospital is unwilling to pay a lot for an orphan drug as the industry did not sustain the cost of the research.

Maria Teresa Baldini then turned the attention to hospital budget. She focused on the cost of new melanoma drugs and the general cost of innovation. In addition, personalised medicine will have an increased cost for any National Health Service (NHS). The Lombardy Oncology Network provides guidelines for treatment and follow-up of the patients with a regional program for melanoma, or a ‘melanoma unit’.

### Second session

The second session contained an additional six talks looking at similar logistical issues in melanoma treatment. First, Giuseppe Pelicci from the IEO looked further at the cost and benefits of drug development. In particular, he looked at the development of molecular drugs or drugs that block cancer growth by interfering with specific molecules. These drugs have been available for about 10 years now and when used are extremely successful. However, molecular drugs only account for about 1% of cancer treatment. In order for molecular drugs to be effective in more cancers, a better idea of biological makeup of tumours is needed as well as novel molecular targets and markers. The International Cancer Genome Consortium catalogues somatic mutations in 500 tumours in 50 different cancer types and/or subtypes and makes the information available. We are learning that cancer is very heterogeneous, containing different groups of cells with different genetic histories, and eventually, it will be considered more of a segmented disease, like an orphan disease. If this is true, then the cost of tailored drugs could become unattainable. Dr Pelicci also talked about developing new drugs and the path to approval. He stated that, in the current system, the academic researchers, pharmas, biotechs, and academic hospitals are too disjointed, and more integration between these groups, as well as regulatory and political authorities, is necessary. In addition, academic research and hospitals should be more involved with drug discovery and development.

In the next talk, Paolo Foggi outlined what the Italian Medicines Agency, AIFA, is doing to improve access to and availability of drugs. Drugs are becoming more expensive, and the need for them is increasing with people living longer and also living with cancer longer. This means that more drugs are needed in the lifetime of any one patient [11]. The aims of AIFA are to guarantee access to medicines, ensure unity of NHCS with regional authorities, grant rapid access to drugs for rare diseases, and provide drug expenditure governance. The challenges of drug access were mentioned, and a number of suggestions on how to improve the situation were given. AIFA is working on a national and regional level to ensure that the pricing of drugs is transparent among many other initiatives to improve the cost/benefit of drugs.

Giorgo L Colombo from the University of Pavia and part of Studi Analisi Valutazioni Economiche stressed the need for new tools to evaluate and help drive discussion around new drugs and technology. Economic evaluation is such a tool that can be used to assess the practicality of treatment options. It compares costs (resources consumed) versus outcomes (clinical, economic, and humanistic) from interventions (pharmaceuticals, non-drug therapies, public health programmes, etc.) pooling together as much information as possible about a treatment scenario. This type of evaluation is something that is done by hospital managers, industry, or government researchers, not just economists. Dr Colombo stressed that the focus of economic evaluation is not to reduce costs, but to find the most efficient use of resources.

The management of new melanoma drugs in Italian hospitals was the topic of the next talk by Francesco Paganelli from the Istituto Oncologico Veneto IRCCS in Padova, Italy. The talk looked specifically at the cost of ipilimumab for all patients in the Veneto Region of Italy as an example for reducing costs. A number of cost-reducing methods were discussed: drug days, rounding doses, the AIFA web-based register, and revaluation. In addition, in his particular example, all patients in the Veneto Region receive treatment at the same hospital, the I.O.V. Melanoma Unit, at the Venetian Oncology Institute. The general idea is that centralisation helps to reduce costs. Paganelli also looked at vemurafenib and noted that centralisation is not as key with this drug because it is given as tablets, so there is no wastage of the drug. In this case, economic burden is calculated by a budget impact analysis.

Finally, Emanuela Omodeo Salè from the IEO discussed new pharmacological therapies for melanoma, which include ipilimumab and vemurafinib. The drugs are expensive and the sustainability over the long term must be verified. In order to save on ipilimumab in the IEO, they administer to all patients on the same day (1 vial costs €2684). In the discussion, the difference in approach between the Veneto region, as presented by Dr Paganelli and the Lombardy region, as presented by Dr Sale, was noted. Veneto had centralised all of their cancer patients, while this was not necessarily the case in Lombardy. It was not clear which method was more efficient or if there was a need to choose one over the other.

### Third session

The first half of the third session was from the perspective of pharmaceutical companies. Cosimo Paga from Bristol-Myers Squibb started off with a talk about immune-oncology, or more specifically, the mode of action of ipilimumab and other drugs. He discussed how combinations of drugs performed and how long patients will be disease free or continue to improve after discontinuation of drug. It was shown that immune-oncology drugs may perform differently from other treatments and that multiple measures may be needed to achieve optimum performance of the drugs.

Luisa Veronese from F. Hoffmann-La Roche Ltd started her talk acknowledging that patients, payers, physicians, and industry all want the same thing; better treatments available as quickly as possible. Roche is a research-focussed organisation that develops pharmaceuticals alongside the development of companion diagnostic tests. She gave a brief summary on the mode of action of vemurafenib through BRAF inhibition. Roche has also worked on overcoming resistance to BRAF inhibition. They have tried ‘targeted combinations’ in which they combined vemurafenib with a number of other drugs to test the usefulness.

Axel Hoos from GlaxoSmithKline (GSK) spoke about their work on combination therapies or their immunotherapies from discovery to pipeline. There are two possible approaches to combination therapy. In the first, the two drugs or treatments will work along the same mechanism or pathway. However, drugs that work along different pathways may be able to do more. GSK is currently collaborating with six other companies which have developed drugs that may combine well with drugs developed by GSK. For success in combination development, there is a significant amount of regulatory work as well as operational discipline [12].

Laurent Thibaut from Bioalliance gave a brief overview of the progress made with their melanoma drug AMEP. They have observed that a combination of Plasmid AMEP with electrotransfer enhances the penetration and thus effect of AMEP. This leads to a longer inhibition of melanoma growth. Toxicity and phase I studies were both promising, and further study of this drug is warranted.

Alfredo Romano from the Celgen Corporation finished the talks from the pharmaceutical companies by sharing some clinical results from work with their drug, nab-Paclitaxel Abraxane, the first true nanotechnology pharmaceutical product to be approved and marketed. This drug has already been successful with breast, non-small-cell lung carcinoma, and pancreas cancers, and is in phase III trials for melanoma.

Ruggero Ridolfi from the Immunoterapia e Terapia Cellulare Somatica IOR Meldola started the second half of the third session with a switch back to strategic issues in his talk about implementing ‘drug days’ in an Italian hospital to cut costs and save waste of the drug. The cost analysis of the drug days showed that about €8000 was saved for every patient, which added up to about €350,000 per year.

Next, Claus Garbe from the University Department of Dermatology from Tübingen, Germany, discussed the cancer centre certification program in Germany. Quality assurance is essential in this process, and centres must fill in a questionnaire have yearly visits from reviewers. Within each centre, interdisciplinary care is standard, with social and psychological care of the patients considered as well. In the area of melanoma, they have produced the S3 (evidence based) Melanoma Guidelines [13]. The guidelines developed after being reviewed and discussed over 3 years by experts from 32 different disciplines of the German Cancer Society. It contains 124 recommendations and statements over 116 pages with 717 citations. The certification programme developed by the Germans stands as an example for uniform care and quality assurance.

Alberto Costa, Scientific Director and CEO of the European School of Oncology (ESO) concluded the third session with a brief discussion on education standards in Western and Eastern Europe. The ESO was established in the 1980s to address the issue of cancer mortality due to lack of awareness, not lack of research. He stated that, in some countries, 20% of patients are dying from cancer, not because of lack of treatment, but because their disease was diagnosed too late or because their region was not able to provide the best treatment. It is necessary to educate the public and also improve the knowledge of cancer doctors and nurses.

## Summary of parallel workshops

The end of the first day and the second day was spent in parallel workshops covering the three main themes of the conference: strategic, clinical, and scientific issues within melanoma care. Each talk lasted approximately 10–15 min, and each workshop was followed by a half an hour discussion, which were summarised by some attendants. The summaries were then presented to the full conference for further discussion.

### Highlights from the strategic workshops

Summarised by Bettina Ryll and Mossimo Monturano, the strategic workshops started off with a discussion on how patients decide where to be treated. Hospitals and centres need to create some sort of qualification or quantification for excellence, which can help patients decide. This could also help patient advocates direct patients where to go. The discussion then switched to health economics. The time where we can afford to pay for anything, new drugs, new treatments, etc., is coming to an end. We have limited resources, and we need to learn how to allocate them in a fair and direct way. There is now a need for more value-based medicine. We must identify what is valuable to us and how to incorporate it into our financial system.

The workshop continued with some specific examples of new treatments. For example, there is a new melanoma treatment using electrochemotherapy and limb perfusion. While the treatments are effective, there are difficulties concerning reimbursement and how to grant access to the treatments. There are also strong regional and national differences that influence what drugs and treatments are used where. Some treatments are more popular in some countries, but not others. Another talk during this workshop focussed on a new development, the frontiers of national NHS. The workshop attendees heard about the directive and goals of this group. The idea behind it is that the patient is free to choose their treatment from anywhere within Europe. However, this idea comes with a number of challenges, including that health systems within Europe are national and how the costs should be covered. Also, it has been shown that patients do not like to travel too far for treatment and are rarely willing to leave their country. To overcome this, a European reference network has been suggested, where doctors and patient advocates can direct patients.

The strategic workshop ended with comments about patient involvement in the decision-making process, the expected increase in molecular therapies due to personalised treatments, and the cost of providing treatment. The group also agreed that it was very useful to have such a varied group of people at this conference and they hoped that the meeting would be a catalyst for improved integration between the various contributors to cancer care.

### Highlights from the scientific workshops

The scientific workshops were summarised by Massimo CP Barberis and Maria Rescigno and contained contributions from pathology, molecular biology, basic research, immunology, and the impact of immunotherapy on melanoma. One point raised during this session was the need for a critical mass in diagnosis and treatment. Both the diagnosis and treatment of melanoma are becoming more specialised, and so by creating specialised centres with larger groups of patients, those involved in diagnosis and treatment will have better knowledge and experience. Such specialised centres would allow for greater levels of interdisciplinarity and integration. Indeed, it was also noted that a greater degree of integrated schemes of treatments was needed and that there should be more multi-centre and multi-disciplinary studies.

The scientific workshop also discussed the challenges involved with the high degree of regulation for new drugs or treatments. Europe has good manufacturing practice guidelines for the development of new drugs, which mandates a very high standard. Institutions with a low budget often find it difficult to maintain the high protocols needed to develop drugs, and oftentimes, by the time the procedures have been approved, the drug is no longer the best option and the researchers need to start again. Efforts should be made to make the process faster.

### Highlights from the clinical workshops

Giuseppe Spadola and Beatrice Colombo summarised the clinical workshops. It started off with a talk on the development of a melanoma network in the Veneto cancer network. Such a network would allow carers to share information on the diagnosis and treatment of melanoma and also to better inform a central referral centre.

The workshop also contained a few presentations on the clinical aspects of diagnosis. Digital follow-up and digital tomography were discussed. It was noted that digital follow-up is the most important for patients who have a high chance of developing a second melanoma. It was also suggested that general practitioners be involved with this digital screening for the second examination. In another talk, the benefits of confocal microscopy were pointed out. Typical histological examinations require a surgical intervention, but confocal microscopy is a non-invasive tool that can help with diagnosis *in vivo*. Surgery of melanomas at different stages was also discussed. Many aspects of surgery in melanoma, including the surgery of the primary, how much is enough, and what to do with positive sentinel nodes, are still debated.

This workshop also looked at the role of the patient and their state of mind. Follow-up strategies in Germany and other countries were considered, and it was noted that different countries will have different priorities based on the social, medical, and economic aspects of the country or region. The benefit of psycho-oncology for both the doctor and patient was also considered. A psychologist can try to help the doctor understand how a patient will react and will help the patient understand and accept a new situation and the clinical aspects of it. Psychological education also becomes an emotional support for the patient.

## Conclusions

The goal of the first meeting of the MIB was to bring together experts from various areas of cancer treatment and to start a dialogue between them. Researchers from industry, academia, government, patients, politicians, and those in charge of budgets and organisation all have different perspectives and priorities when it comes to the development of new therapies. Despite their differing points of view, all agree that it is essential to get the best treatments to the most patients as quickly as possible. However, such a goal is limited by sustainability and the ever-increasing cost of treatments. The group hopes to use their dialogue to find a balance between these two issues.
